# Congenital aortocaval fistula combined with patent ductus arteriosus in an infant: a case report

**DOI:** 10.1093/icvts/ivac260

**Published:** 2022-10-29

**Authors:** Xianting Jiao, Liqing Zhao, Yurong Wu, Sun Chen

**Affiliations:** Department of Pediatric Cardiology, Xinhua Hospital Affiliated to Shanghai Jiao Tong University School of Medicine, Shanghai, China; Department of Pediatric Cardiology, Xinhua Hospital Affiliated to Shanghai Jiao Tong University School of Medicine, Shanghai, China; Department of Pediatric Cardiology, Xinhua Hospital Affiliated to Shanghai Jiao Tong University School of Medicine, Shanghai, China; Department of Pediatric Cardiology, Xinhua Hospital Affiliated to Shanghai Jiao Tong University School of Medicine, Shanghai, China

**Keywords:** Aortocaval fistula, Patent ductus arteriosus, Percutaneous interventional therapy

## Abstract

Congenital aortocaval fistula (ACF) is a rare cardiac malformation. While it can occur in combination with patent ductus arteriosus (PDA), this has not been reported. In this case, a 1-year-old infant had a heart murmur found in a routine physical examination, and PDA was revealed by transthoracic echocardiography and abdominal ACF was detected by three-dimensional coronary artery computed tomography. Percutaneous interventional therapy, used for ACF and PDA, was performed to occlude the malformation. The patient presented good health without any discomfort at a 1-year follow-up. The percutaneous closure of ACF and PDA with an Amplatzer vascular device can be considered an appropriate option.

## INTRODUCTION

Aortocaval fistula (ACF) is a rare condition that is both observed as an acquired and congenital condition and was first described by Syme in 1831 [1]. Congenital cases are rarely reported and have poorly defined characteristics with uncertain pathogenesis [2]. However, its occurrence in combination with patent ductus arteriosus (PDA) in children has not been reported.

## CASE PRESENTATION

**Figure 1:**
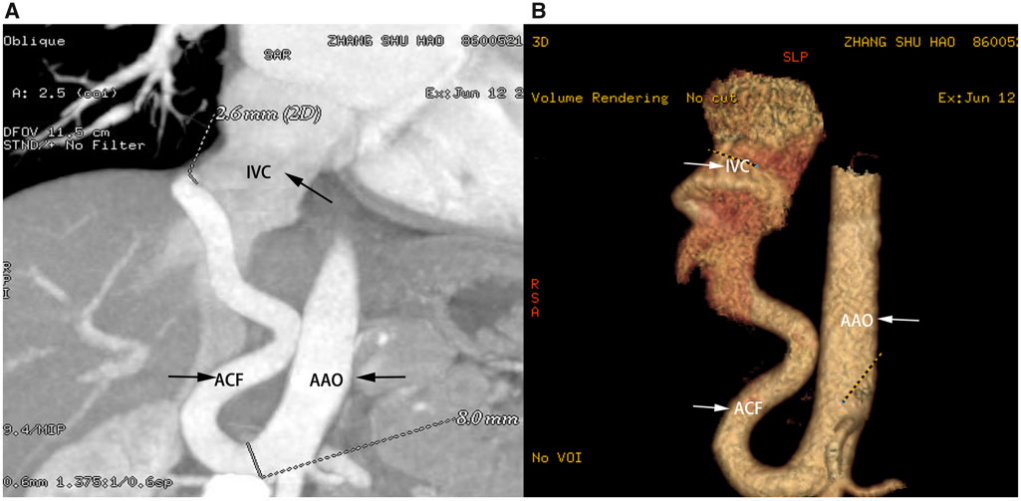
Computed tomography angiography showing a tortuous, large, and very long fistula between the abdominal aorta and inferior vena cava. IVC: Inferior vena cava; AAO: Abdominal aorta; ACF: Aortocaval fistula.

A 1-year-old infant had a heart murmur found on routine physical examination. The PDA was first revealed by transthoracic echocardiography. Furthermore, abdominal arterio-venous malformation was detected by three-dimensional coronary artery computed tomography. The proximal end of the abdominal aorta was widened by ∼11.0 mm, and a large vessel with an inner diameter of 7.1 mm could be seen on the right side of the distal opening of the abdominal trunk. It contorted to the right side and entered the inferior vena cava IVC near its insertion into the right atrium, with an opening of ∼2.6 mm (Fig. 1A and B).

**Figure 2:**
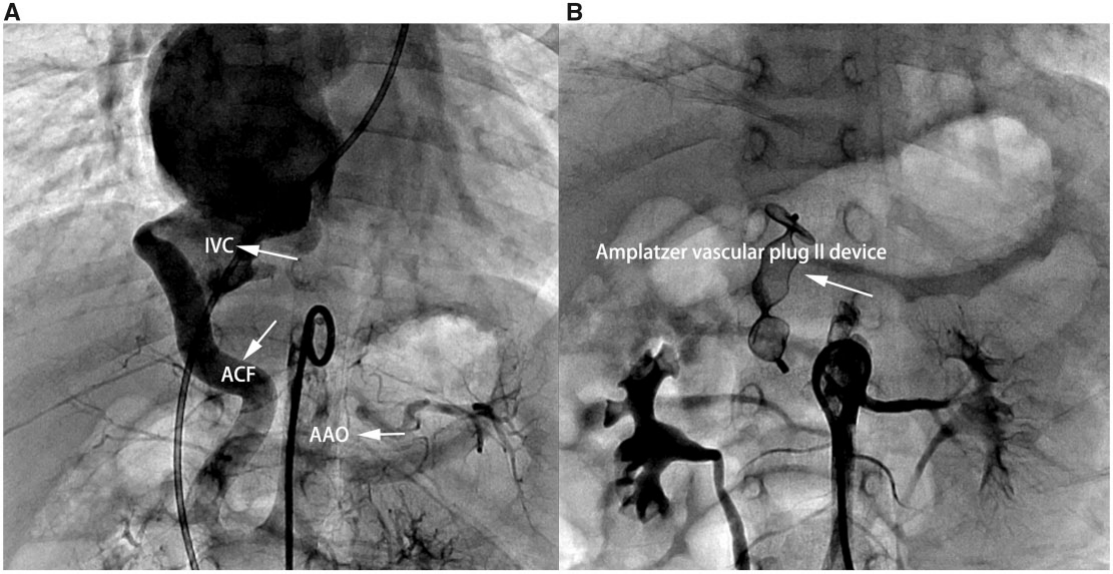
Aortography showing the distal aortocaval fistula distal. (**A**) Notice the dilatation of the inferior vena cava. (**B**) Aortography showing complete occlusion of the fistula with a 12-mm Amplatzer vascular plug II device.IVC: Inferior vena cava; AAO: Abdominal aorta; ACF: Aortocaval fistula.

We adopted the transcatheter method to close the fistula. Descending aorta angiography confirmed the diagnosis of PDA and an unusual tortuous fistula between the AA and IVC (Fig. 2A). Further a 12-mm Amplatzer vascular plug II device (AGA Medical Corporation, Golden Valley, MN, USA) was loaded into the catheter, and the distal skirt of the device was placed on the narrow and curved part, 5–6 mm away from the proximal fistula. A non-selective contrast agent was injected into the abdominal aorta, which revealed occlusion of the fistulous tract with the Amplatzer vascular plug IV device in a stable position at the proximal mouth of the fistula (Fig. 2B). PDA was also closed with the catheter using a 4–6-mm Amplatzer Duct Occluder-II device. No adverse clinical events were reported at the 1-year follow-up (Table 1).

**Table 1:** The relevant literature presentation

Authors	Reporting year	Age	Clinical symptoms	Physical examination	Malformations portion	Treatment protocols	Follow-up time
Sedat Giray Kandemirli *et al.*	2019	3 years	/	Continuous murmur over the left sternal border	Descending aorta and the left brachiocephalic vein	/	
Michael *et al.*	2001	8 weeks	Intermittent tachypnoea, congestive heart failure	Grade 2/6 continuous murmur	Descending thoracic aorta and the the azygous vein and the innominate vein	Coil occlusion	1-Year follow-up
Osman Baspinar *et al.*	2005	15 months	No symptoms	An intensive continuous murmur in the left paraspinal region	The descending thoracic aorta to the hemiazygos vein	No therapy	1-Year follow-up
David Sutton *et al.*	1977	5 years (Case 1)	No symptoms	Continuous murmur	The aorta and the hemi-azygos system	No operation	Regular follow-up
David Sutton *et al*.	1977	10 years (Case 2)	No symptoms	Continuous murmur	Intercostal aorta and the azygos system	Surgical operation	Regular follow-up
Fahrettin Uysal *et al.*	2015	5 years	No symptoms	Continuous murmur heard in the right hemi-thorax	The mid-thoracic aorta and the azygos vein	Amplatzer Vascular Plug-2 device	1-Year follow-up

## DISCUSSION

Congenital ACF is a rare condition with unclear embryological pathogenesis. Anatomical characteristics are variable, and every fistula is unique, which was found in case reports and small studies.

In our case, physical examination revealed a continuous murmur. Computerized tomography can provide important information regarding the extent and anatomy of aorto-venous fistulas. The pathophysiology and haemodynamic consequences of ACF are caused by circulation through the high-resistance arterial circuit to the low-resistance venous circuit [3]. To date, congestive heart failure caused by high-output status is well documented in patients presenting arterio-venous fistulas [4]. More evidence has shown that the use of endovascular techniques is an attractive therapeutic alternative to surgical procedures for ACF [5]. Percutaneous transcatheter closure is an effective and appropriate method for both ACF and PDA patient treatment.

In conclusion, congenital ACF is rare, and this is the first report of concurrent PDA. The percutaneous closure of ACF and PDA with an Amplatzer vascular device is safe and effective.
